# Identification of *Francisella tularensis* Cluster in Central and Western Europe

**DOI:** 10.3201/eid1512.080805

**Published:** 2009-12

**Authors:** Paola Pilo, Anders Johansson, Joachim Frey

**Affiliations:** University of Bern, Bern, Switzerland (P. Pilo, J. Frey); Umeå University, Umeå, Sweden (A. Johansson); Swedish Defence Research Agency, Umeå (A. Johansson); 1 These authors contributed equally to this work.

**Keywords:** Francisella tularensis, Switzerland, genetic analysis, erythromycin susceptibility, bacteria, dispatch

## Abstract

We conducted a molecular analysis of *Francisella tularensis* strains isolated in Switzerland and identified a specific subpopulation belonging to a cluster of *F. tularensis* subsp. *holarctica* that is widely dispersed in central and western continental Europe. This subpopulation was present before the tularemia epidemics on the Iberian Peninsula.

Tularemia is a classical zoonosis caused by the facultative intracellular bacterium *Francisella tularensis*; it is transmissible to humans at infectious doses as low as 10–50 bacteria when inhaled in aerosols or by inoculation of the skin. Traditionally, tularemia is thought of as a disease contracted by persons performing outdoor activities such as hunting or farming, but it can also be acquired from pets, for example, hamsters or prairie dogs, which are occasionally traded internationally (*1*). During the past 15 years, the reemergence of tularemia has been reported in several European countries (*2*–*4*). Spain is a notable example, reporting 916 human infections from 1997 through 2007 in the Castilla and León regions alone (*5*). However, tularemia is rarely diagnosed in central Europe. In Switzerland, *F. tularensis* infection was first described in the 1950s, but the pathogen was not isolated until 1996, when *F. tularensis* infection began to reappear sporadically. To better understand the genetic diversity of Swiss *F. tularensis* strains and their relationship to strains from other geographic areas, we analyzed strains from Switzerland by using several methods that had been previously demonstrated to resolve genetic differences between *F. tularensis* subsp. *holarctica* strains: multilocus tandem repeat analysis (MLVA), canonical *F.* *tularensis* insertion deletion element (Ftind) analysis, and region of difference (RD) 23 analysis (*3*,*6*,*7*).

## The Study

**Figure 1:**
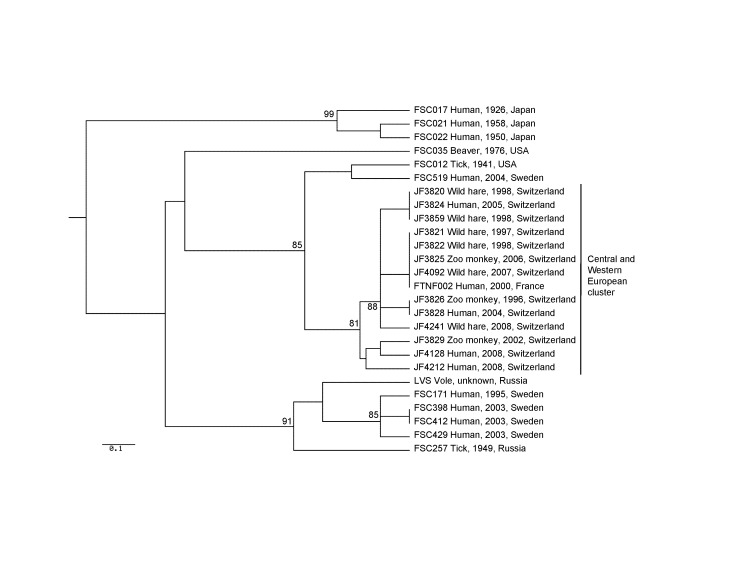
Genetic relationships between *Francisella tularensis* subsp. *holarctica* strains isolated in Switzerland and strains of wider geographic origin. The unweighted pair group method with arithmetic mean phylogram is based on the combined Ftind and multiple-locus variable-number tandem repeat analysis. Bootstrap values >80% are given at the respective nodes and were calculated by using 10,000 iterations. Scale bar indicates genetic distance.

Thirteen *F. tularensis* isolates collected over the past 10 years in Switzerland (Figure 1) were subjected to extensive genetic characterization. The species and subspecies designations of all strains were confirmed by real-time PCR that targeted the *fopA* gene and by amplification of the RD1 region (*8*), which showed that all strains were *F. tularensis* subsp. *holarctica*. A reference panel of 12 *F. tularensis* subsp. *holarctica* strains (*7*) and the genome sequence of the strain isolated in France, FTNF002 (GenBank accession no. NC_009749), were included in the study to represent the currently known genetic subpopulations within the subspecies. All strains from Switzerland were genetically characterized at 6 highly variable loci (by MLVA) and 14 more stable loci that indicate the classification *F. tularensis* subsp. *holarctica* strains into genetic subpopulations (by Ftind analysis) (*3*,*6*,*7*). The RD analysis was also performed because a 1.59-kb deletion marker, RD23, was reported to be restricted to strains from France and Spain (*3*). The MLVA markers (M3, M6, M20, M21, M22, and M24) and Ftind markers (Ftind 25–38) were amplified by PCR and then sequenced with an ABI Prism 3100 genetic analyzer (Applied Biosystems, Foster City, CA, USA) and the BigDye Terminator cycle sequencing kit (Applied Biosystems). DNA fragment sizes were calculated from the nucleotide sequences of the MLVA and Ftind markers and used to compare the isolates with previously analyzed strains from the United States, Japan, France, and Russia (*3*,*7*). The RD23 marker was assayed by using standard PCR and agarose gel methods as previously described (*3*). A cluster analysis based on the MLVA and indel size data was performed by using BioNumerics version 3.5 (Applied Maths, Kortrjik, Belgium).

As expected, the indel markers served to place each strain into major branches of the cluster tree, and the more variable MLVA markers provided the fine resolution at the tips of the tree. The Switzerland strains belonged to the same genetic cluster as the *F. tularensis* strain FTNF002 from France (Figure 1) that in a previous work clustered with strains from Spain (*3*,*9*). Moreover, all the Swiss strains exhibited the 1.59-kb genomic deletion at the RD23 locus and the unique 464-bp size at MLVA marker M24, which confirmed their close relationship to the French strain FTNF002 as well as to other strains from France and Spain (*3*).

**Figure 2:**
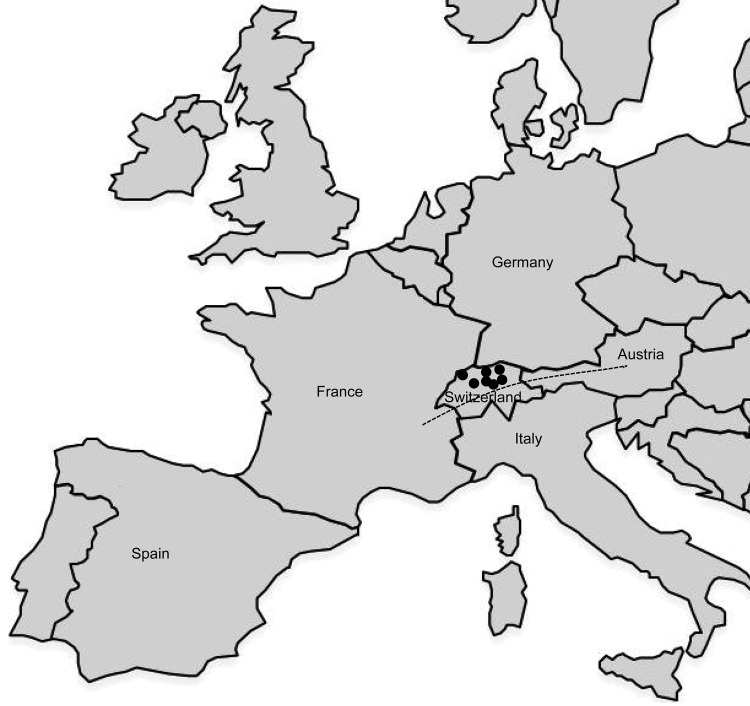
Geographic distribution of *Francisella tularensis* subsp. *holarctica* strains of the central and western European genetic cluster isolated in Switzerland. Dots represent the geographic origin of the isolates (from 7 Swiss cantons). The dashed line indicates the Alps. Strains of the subclade B.Br:FTNF002–00 are known to be present in France and Spain.

The finding of *F. tularensis* strains in Switzerland represent sporadic occurrences of tularemia without any obvious epidemiologic connection. The strains originated from 6 hares, 3 monkeys, and 4 persons and were collected at different locations in Switzerland over a period of 10 years (Figure 2). The human infections most likely occurred through direct contact with wild animals, through rodent bites, and through consumption of a hare cooked at low temperature. Those isolates could be resolved into 7 different genotypes (Figure 2). Four Swiss strains displayed a genetic profile identical to that of the representative French strain FTNF002 (*3*). The other 6 genotypes were closely related to FTNF002, and all corresponded to the subclade B.Br:FTNF002–00 as defined by canonical single nucleotide polymorphisms by Vogler et al. (*9*). This cluster, which also contained the strains from the Iberian Peninsula, seems to have spread throughout central and western Europe. Moreover, all the Swiss strains were susceptible to erythromycin (MICs 0.25 μg/mL to 1 μg/mL), which is a phenotypic marker that has previously been suggested to divide *F. tularensis* subsp. *holarctica* strains into 2 taxonomic groups (*10*–*12*).

## Conclusions

Strains of *F. tularensis* from Switzerland (central Europe) genetically clustered with strains from France and Spain (western Europe) as determined by the unique 464-bp genetic marker M24 and a specific deletion at marker RD23. Furthermore, strains within the cluster differed at only 2 MLVA markers,and 4 other MLVA and 14 Ftind markers were identical. In a previous study that included strains from the 1997–1998 tularemia outbreak in Spain, the specific M24 allele and the RD23 deletion were found in 49 of 49 strains from Spain and France but in only 1 of 189 strains from 7 northern and eastern European countries and Japan (*3*). The tularemia outbreak of 1997–1998 in Spain, which resulted in >500 human cases (*5*), was thus caused by *F. tularensis* strains that were genetically closely related to strains recovered in Switzerland from 1996 onwards, before the beginning of the outbreaks in Spain. This genetic relationship shows that factors other than the presence or introduction of a specific clone of the infectious agent per se determined the magnitude of the tularemia outbreaks in Spain. For epidemiologists to understand the distribution of *F. tularensis* (and other rare disease agents) in the environment and their propagation across national and geographic borders, surveillance programs that include molecular analyses of these agents should be undertaken in multiple countries, and the resulting data should be shared internationally.
